# Gender Bias in Clinical Trials of Sodium–Glucose Cotransporter 2 Inhibitors for the Treatment of Type 2 Diabetes Mellitus: A Systematic Review

**DOI:** 10.1155/jdr/3733178

**Published:** 2025-04-22

**Authors:** Santiago José Lora-Escobar, Lupe Rodríguez-de Francisco, Manuel López-Feijoo, Bernardo Santos-Ramos, Virginia Bellido, Rosa Casado-Mejía, Héctor Acosta-García

**Affiliations:** ^1^Pharmacy Department, Hospital Universitario Virgen del Rocío, Seville, Spain; ^2^Endocrinology and Nutrition Department, Hospital Universitario Virgen del Rocío, Seville, Spain; ^3^Equality Unit, University of Seville, Seville, Spain; ^4^Faculty of Nursing, Physiotherapy and Podiatry, University of Seville, Seville, Spain

## Abstract

**Aims:** The study is aimed at assessing gender bias in Phase III clinical trials (CTs) of sodium–glucose cotransporter type 2 (iSGLT2) inhibitors in adults with Type 2 diabetes mellitus (T2DM).

**Methods:** We searched PubMed and EMBASE databases until June 2023. To estimate sex bias in recruitment, the difference between F-Particip (fraction of women recruited) and F-Prev (prevalence fraction of women with T2DM) was calculated. A significant sex bias in recruitment was considered to exist when the difference between F-Particip and F-Prev was greater than 0.05, 0.1, and 0.2. The analysis was considered to have a sex bias when the efficacy variables were not analyzed by sex. Gender of the first, last, and corresponding author was also assessed.

**Results:** In total, 70 articles met all inclusion criteria. Sex bias in recruitment showed variable results depending on the reference value. No significant sex bias in recruitment was found in the total number of included patients. Examining each CT individually, using values of significant sex bias in recruitment of ±0.05, ±0.1, or ±0.2, we found that 41.4%, 20%, and 5.7% of the trials, respectively, showed this bias. In 34.3% of the articles, women were the first, last, or corresponding authors. Sex-based analysis of the results was performed in five studies.

**Conclusions:** Although the proportion of women included in iSGLT2 CTs seems acceptable, gender bias persists in the analysis of variables and in the study authors. However, the lack of gender focus may be explained by the characteristics of the patients included in the CTs.

## 1. Introduction

Gender bias is a widely recognized term in medical research that highlights the lack of equal inclusion and the absence of sex-specific analyses in clinical trials (CTs). Historically, women have often been excluded from CTs showing underrepresentation relative to men [1, 2]. In response to the acknowledgment of gender bias in CTs, national and international organizations have initiated legislative endeavors to enhance female inclusion [2]. The Revitalization Act of 1993, enacted by the National Institutes of Health (NIH), laid down legal mandates and directives for researchers to guarantee the inclusion of women as well as men in CTs and to analyze their results by gender or sex [3]. The US Food and Drug Administration (FDA), responsible for overseeing the approval of all prescription drugs and devices, has introduced an Action Plan which will integrate the examination of gender-based differences into the drug and device approval processes [4]. The EU has been less sensitive to these issues, and until 2006, gender considerations were not taken into account in research, albeit in a limited way [5]. In recent years, there has been an increasing awareness, as evidenced by, for example, the first Horizon Europe Strategic Plan (2021–2024) [6], which includes two new developments related to this theme: (1) The analysis of sex and gender in research design is required in all applications, unless its irrelevance is justified, and (2) institutions must implement a Gender Equality Plan (GEP) in order to be eligible for funding from the European Commission. However, these measures have not been sufficient, as gender bias persists in medical research.

In this regard, the study by Feldman et al. claims that not enough progress has been made on this issue [3]. In 120 randomized CTs across 12 specialties involving a total of 160,801 participants, only 24.6% were women. In a literature search from 2000 to 2002, the ratio of trial participants to cancer cases in the population was lower in women than in men in both colorectal and lung cancer trials. Between 1999 and 2018, 13 analyses for sex bias in clinical studies were identified [3]. Xiao et al. demonstrated that the gender of the author can impact the gender composition of participants involved in surgical studies [7].

Some authors suggest several reasons why women are less included in studies. Steinberg et al. state that the reason was to ensure the homogeneity of treatment effect and reduce potential maternal–fetal liability [2]. In addition, Van Diemen et al. performed an extensive literature search; the results of which show that women declined to participate in CTs more often than men. The perception of harm and transport problems were the main reasons [8]. Female researchers seem to exhibit greater sensitivity regarding this topic, as it has been observed that they tend to include a higher proportion of women compared to male researchers [7].

This study focuses on CTs of patients diagnosed with Type 2 diabetes mellitus (T2DM). Globally, an estimated 17.7 million more men than women are diagnosed with diabetes mellitus (DM) [9]. The predisposition to and development of the disease in both sexes depend on physiological, genetic, lifestyle, and socioeconomic factors [10]. By contrast, gender bias in clinical practice influences the diagnosis and management of the disease. At diagnosis, men are more often diagnosed at younger ages compared to women [11]. However, at the time of diagnosis, women appear to have greater exposure to risk factors such as obesity and psychosocial stress [9, 10]. They are also exposed to greater hormonal fluctuations, due to reproductive factors such as pregnancy, leading to gestational diabetes, another risk factor for progression to T2DM [9]. Skvortsova et al. reported that “women with diabetes have worse quality of life, worse mental well-being and more daily limitations compared to men with diabetes” [11]. Moreover, the risk of cardiovascular mortality is influenced by T2DM, affecting women and men unequally [9]. Women with T2DM have higher glycosylated hemoglobin (HbA1c) and low-density lipoprotein (LDL) cholesterol levels relative to men, which are important risk factors for cardiovascular complications [11]. T2DM triples the risk of cardiovascular mortality in women and doubles this risk in men [10, 11]. Regarding pharmacotherapeutic management, more optimal therapies are often documented in diabetic men than in diabetic women [11, 12]. Men tend to receive antidiabetic treatment based on combined oral drugs, angiotensin-converting enzyme (ACE) inhibitors, and lipid-lowering medications [13]. Furthermore, women with diabetes are less likely to receive sodium–glucose cotransporter 2 (SGLT2) inhibitors, cardioprotective and renoprotective drugs. This discrepancy in treatment may have significant clinical consequences; SGLT2 inhibitors significantly reduce deaths from cardiovascular conditions, hospitalizations for heart failure, and progression of kidney disease among patients with Type 2 diabetes [12].

As we have pointed out above, the integration of sex and gender analysis in biomedical research is essential for ensuring high-quality scientific research and for translating these findings into clinical practice [14]. This systematic review is aimed at assessing gender bias in the design and analysis of the results of published Phase 3 CTs of SGLT2 inhibitors for the treatment of T2DM.

## 2. Materials and Methods

### 2.1. Eligibility Criteria

This systematic review was conducted in accordance with the Preferred Reporting Items for Systematic Reviews and Meta-Analyses (PRISMA) Equity 2020 Extension [15]. The review protocol was registered in the International Prospective Register of Systematic Reviews (PROSPERO) Database (Registration Number CRD42022359480).

Inclusion criteria were as follows:

− Patients over 18 years of age diagnosed with T2DM.
− Antidiabetic drugs from the iSGLT-2 group or combinations of these and other antidiabetic groups.
− Phase III CTs with a placebo control group or another antidiabetic drug.
− CTs analyzing diabetes-related outcomes.

We excluded the following:

− Extension and post hoc studies.
− Short reports, letters to the editor, papers, and communications to congresses.
− CTs in which the results are related to cardiovascular morbidity.

### 2.2. Information Sources and Search Strategy

We performed a literature search in Medline—through the OvidSP platform—and Embase, until 28th June 2023. The complete search strategies used are shown in Table 1.

### 2.3. Study Selection

A peer review of eligible articles was performed. Disagreement was settled by consensus or by a third investigator.

### 2.4. Data Collection and Analysis

Three reviewers independently extracted data and examined all extraction sheets to ensure their accuracy. The following study characteristics were recorded as independent variables:

− Drug under study.
− Publication year.
− Objective of the CT (efficacy/safety).
− Total number of patients recruited.
− Average age of patients recruited.
− Location of the trial: United States, Europe, Japan, Australia, rest of the world (ROW), or international trial.
− Study arms of the CT.
− Financing of the CT: pharmaceutical industry or independent (trials were considered to be promoted by the pharmaceutical industry if one of the authors was employed by a pharmaceutical company or if direct funding was specified).

For the analysis of sex and gender bias, the SAGER guidelines and the FDA guide were used [16]. The following variables were also recorded:

− Total number of women included.
− Gender of the first, corresponding, and last author.
− Whether or not there are sex-stratified results for the main efficacy outcome.
− Whether or not there are sex-stratified results for the secondary efficacy outcomes.
− Whether studies described pregnancy as an exclusion criterion.
− Whether they included women using hormonal contraceptives and analyzed the interaction between hormonal contraceptives and the study drug.
− Whether there was a study of the influence of the phase of the menstrual cycle on the pharmacokinetics and response to the drug.

The main outcomes analyzed were as follows:

− Sex bias in recruitment: estimated using the female prevalence fraction (F-Prev) and the female participant fraction (F-Particip). The F-Prev for DM (0.48) was obtained from a database synthesized from multiple data sources, used in the study of Feldman et al. [3]. In this study, diabetes prevalence is calculated by dividing the global morbidity count for female participants in the disease category by the global morbidity count for both male and female participants using data from Global Health Data Exchange (GHDx) [17]. F-Particip was defined as the fraction of female participants among all participants for each study and was estimated by dividing the total number of female participants by the total number of male and female participants of each study. Sex bias in recruitment in clinical studies was defined as F-Particip minus F-Prev (values for sex bias ranged from −1 to 1, with 0 indicating no bias; negative sex bias indicates that female participants were represented less than male participants). A substantial sex bias in recruitment was defined as a calculated sex bias ± 0.05, as in the study of Feldman et al. [3]. We also considered CTs with calculated sex bias in recruitment of ±0.1 and ±0.2.
− Percentage of women among the patients recruited.
− Percentage of CTs in which women were listed as first, corresponding, or last author.
− Percentage of CTs presenting the main efficacy outcome by sex.
− Percentage of CTs presenting the secondary efficacy outcome by sex.
− Percentage of CTs describing pregnancy as an exclusion criterion.
− Percentage of CTs that included women using hormonal contraceptives and analyzed the interaction between hormonal contraceptives and the study drug.
− Percentage of CTs that studied the influence of the phase of the menstrual cycle on the pharmacokinetics and response to the drug.

We also applied a subgroup analysis for the variables: drug; location; date of publication; first, corresponding, and last author gender; and sample size.

## 3. Results

**Figure 1:**
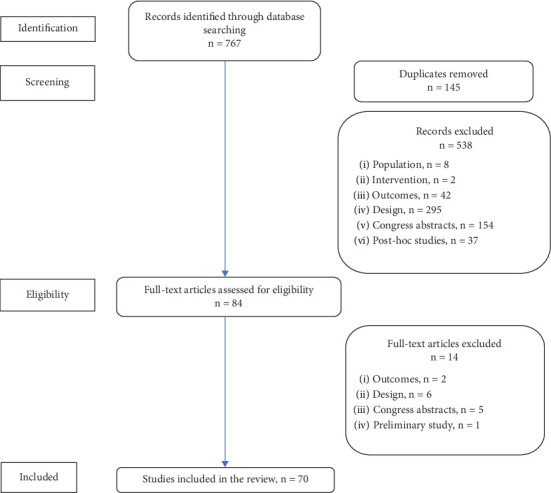
Study selection flowchart.

Our search identified 767 citations, of which 683 were excluded at the initial stage of screening and an additional 14 on full-text review, yielding a total of 70 studies that met all inclusion criteria.  Figure 1 shows the flow of studies through the review process.  Table 2 shows the main characteristics of the included trials by drug, publication date, and author.

Table 3 displays the variables focusing on potential gender bias. The overall representation of women in CTs was 17,275 out of 38,119 included patients (45.3%), indicating no gender bias concerning patient recruitment for the studies (sex bias = −0.027). However, when considering the CTs individually, they do reveal sex bias in 41.4% of the studies (sex bias ± 0.05). Among these studies, 31.4% of the trials demonstrated underrepresentation of women, while 13.8% showed overrepresentation. When a significant deviation of ±0.1 or ±0.2 is taken into account, gender bias in recruitment is observed in 20% and 5.7% of the trials, respectively. The analysis of the results by sex was only conducted in five of the studies [40, 59, 68, 78, 81] and was discussed in only one trial [48]. Of a total of 70 studies, 24 (34.3%) of the articles had a woman as the first, last, or corresponding author. Specifically, 2 (2.9%) had three women in key authorship roles, 11 (15.7%) had two women, 11 (15.7%) had one woman, and 46 (65.7%) had no women in these roles. Furthermore, none of them considered factors such as hormone replacement therapy, hormonal contraceptives, or the menstrual cycle in relation to drug response or pharmacokinetics. Additionally, pregnancy was an exclusion criterion in 35 CTs.

Table 4 shows the subgroup analysis. When examining the data by drug, no statistically significant variances were observed in the representation of the most studied medications, ranging from 33.2% for tofogliflozin to 52.3% for luseogliflozin. Geographical variations were noted in the representation of women across studies. For instance, studies conducted in Europe demonstrated a higher proportion of female participants (57.5%) compared to those in Asia (38.4%). Regarding analyses by sex, it is notable that the five studies that took this factor into account were conducted in Asia. No significant differences were found in the representation of women between studies published from 2010 to 2015 (44.2%) and those published from 2016 to 2023 (46.7%). Noteworthy differences were observed in the representation of women across studies helmed by female authors, whether as first, corresponding, or last authors. Those studies in which women occupied three of these positions exhibited a markedly higher percentage of female participants (53.2%) in contrast to those led by male authors (45.9%) or where two or only one woman was included in the authorship (44.4% and 45.6%, respectively). Additionally, studies with a sample size fewer than 200 patients demonstrated the lowest representation (41.1%), whereas those between 400 and 700 patients tended to have a higher representation of women (47.9%).

## 4. Discussion

The results of our study show that in most CTs, the representation of women is slightly lower than that of men. A substantial female underrepresentation is defined as a calculated sex bias in recruitment ≤ −0.05, as reported by Feldman et al. [3], a study evaluating sex bias across a wide range of diseases. This value taken as a reference could overestimate or underestimate the representation of both sexes. In this regard, when applying different thresholds for significant sex bias (±0.05, ±0.1, or ±0.2), we found that 41.4%, 20.0%, and 5.7% of CTS, respectively, exhibited either an overrepresentation or underrepresentation of women. Therefore, our results confirm that diabetes is not a disease with relevant sex bias in recruitment in CTs, such as cardiovascular diseases or chronic kidney disease [3].

Nevertheless, of all the studies, only five performed an analysis by sex of their results [40, 59, 68, 78, 81], which represents a deep sex bias in the design of CTs. None of these CTs found significant differences in their results in the subgroup analysis by sex, which indicates that the mechanism of action of these drugs is not influenced by gender. However, this lack of differences could be due to an insufficient number of participants or inadequate analysis. Notably, four of these studies included fewer than 300 participants and three had a significant sex bias. Therefore, guidelines recommend the inclusion of this valuable information in published studies [15, 16].

Furthermore, all the included trials were funded by pharmaceutical companies. Accordingly, a scarcity of resources would not be an excuse for the lack of focus on gender aspects. On the other hand, in several studies, we observed that there was an analysis by sex of the incidence of genital infections as a safety event. This is not reflected in our results, as gender stratification of safety variables was not one of the measures analyzed in our review. Another remarkable aspect is that the only articles that analyzed the results by sex were from Asian countries, even though these were the CTs with the lowest representation of women.

Similarly, our review also supports that women are still underestimated in the scientific scene. The underrepresentation of women as first and corresponding authors is a well-described issue in the scientific literature [88, 89]. Around one in three studies included a woman as first, last, or corresponding author. In these, calculated sex bias in recruitment was positive, which may suggest that female researchers in CTs tend to recruit more female participants. This endorses the conclusions of a similar study of sex bias [7]. Nevertheless, principal investigators typically have minimal influence on industry-funded trials, leading us to believe there is not a direct connection between these two occurrences [90]. Moreover, like many other medical specialties, endocrinology has historically been male-dominated, yet the representation of women in the endocrinology workforce is rapidly growing [91]. This is also reflected in our study, since more than 95% of the articles in which women occupy first, last, or corresponding authorship were published between 2015 and 2023.

In our review, no CT took into consideration factors such as hormone replacement therapy, hormonal contraceptives, or the menstrual cycle on the response or the pharmacokinetics related to the drugs in the study. Furthermore, pregnancy was an exclusion criterion in half of the studies. It is probable that in the rest of the CTs, this factor was also a reason for exclusion, even though it was not directly mentioned in the article. All these facts go against the minimum requirements of scientific validity demanded by international guidelines [15, 16]. However, this may be explained by the average age of the patients included in the CTs, which suggests that these factors discussed above may not be relevant.

Previous systematic reviews of gender bias that characterized women's participation in CTs of biological agents for migraine [92] or severe asthma [93] concluded that this population was well represented compared to previous studies [94]. This may be explained by the higher prevalence of these diseases in women.

Therefore, our study suggests that the proportion of women in CTs is increasingly related to their prevalence in each disease. However, as in the reviews mentioned above, our work shows that there is still a great lack of focus on other variables such as the stratification by sex in the analysis of the main and secondary results, the discussion of the results analyzed by sex, and the absence of the concept of “gender” in the text. This is in line with a recent study [95] that highlighted the poor integration of sex and gender in diabetes research. This research also suggests that future efforts should focus on enforcing compliance of CTs with guidelines for sex and gender reporting requirements. In the same way, in a study of sex bias in CTs in gastroenterology and hepatology [96], the authors found that although awareness of the sex gap in CTs has increased, sex equity has not yet been achieved.

To our knowledge, our study is the first systematic review to evaluate the concept of gender and sex bias in the CTs of iSGLT2 authorized in the treatment of T2DM. This is also the first systematic review to quantify sex bias taking into account the prevalence of the disease and not only the representation of women in CTs, using indicators such as F-Prev and F-Particip. Moreover, this systematic review was conducted following the PRISMA Equity 2020 Extension [15]. The systematic search was also performed with no publication date, language, or geographic location restrictions in two of the largest healthcare databases. Our study sample is also considerable, with 70 studies and 38,119 patients included.

The main limitation of our work is that only Phase III CTs were included. These trials are the basis for the approval of new drugs by the regulatory agencies of each country. Accordingly, if gender bias is found in these trials, the results obtained may not be applicable to the target population. Another limitation is that the quality of included studies and risk of bias were not analyzed. This systematic review did not assess drug efficacy, and the quality of the studies should not influence the variables we aimed to analyze. Although this was not a primary objective, such an assessment could have further strengthened the findings of this study.

In conclusion, in the case of iSGLT2, the proportion of women and men in CTs seems to be acceptable, but gender bias persists in the analysis of the variables and in the main authors of the studies. However, although guidelines recommend the inclusion of this valuable information and an appropriate design, the characteristics of the patients included in the CTs may explain the lack of a gender focus.

## Figures and Tables

**Table 1:** Complete search strategy for the different databases.

**Healthcare database**	**Search strategy**
PubMed/Medline	((dapagliflozin.mp.) OR (empagliflozin.mp.) OR (canagliflozin.mp.) OR (bexagliflozin.mp.) OR (ertugliflozin.mp.) OR (remogliflozin.mp.) OR (luseogliflozin.mp.) OR (ipragliflozin.mp.) OR (tofogliflozin.mp.)) AND (Diabetes Mellitus, Type 2/dt [Drug Therapy]) AND [clinical trial, phase iii]/lim
Embase	((“bexagliflozin”:ti,ab,kw) OR (“empagliflozin”:ab,ti,kw) OR (“dapagliflozin”:ab,ti,kw) OR (“ertugliflozin”:ab,ti,kw) OR (“canagliflozin”:ab,ti,kw) OR (“remogliflozin”:ab,ti,kw) OR (“luseogliflozin”:ab,ti,kw) OR (“ipragliflozin”:ab,ti,kw) OR (“tofogliflozin”:ab,ti,kw)) AND (“non insulin dependent diabetes mellitus”/de) AND (“phase 3 clinical trial”/de OR “phase 3 clinical trial topic”/de)

**Table 2:** Characteristics of the included clinical trials.

**Study (first author)**	**Mean age of patients (years)**	**Location**	**Funding** ^**a**^	**Intervention**	**Objectives** ^**b**^
*Bexagliflozin*					
Allegretti et al. [18]	69.6	International	Ph. companies	Bexagliflozin 20 mg/placebo	E + S

*Canagliflozin*					
Bode et al. [19]	63.6	International	Ph. companies	Canagliflozin 100 mg/canagliflozin 300 mg/placebo	E + S
Cefalu et al. [20]	56.2	International	Ph. companies	Canagliflozin 100 mg/canagliflozin 300 mg/glimepiride	E + S
Lavalle-González et al. [21]	55.4	International	Ph. companies	Canagliflozin 100 mg/canagliflozin 300 mg/sitagliptin 100 mg/placebo	E + S
Schemtaner et al. [22]	56.7	International	Ph. companies	Canagliflozin 300 mg/sitagliptin 100 mg	E + S
Stenlöf et al. [23]	55.4	International	Ph. companies	Canagliflozin 100 mg/canagliflozin 300 mg/placebo	E + S
Wilding et al. [24]	56.8	International	Ph. companies	Canagliflozin 100 mg/canagliflozin 300 mg/placebo	E + S
Yale et al. [25]	68.5	International	Ph. companies	Canagliflozin 100 mg/canagliflozin 300 mg/placebo	E + S
Forst et al. [26]	57.4	International	Ph. companies	Canagliflozin 100 mg/canagliflozin 300 mg/placebo	E + S
Inagaki et al. [27]	58.0	Asia	Ph. companies	Canagliflozin 100 mg/canagliflozin 300 mg/placebo	E + S
Ji et al. [28]	56.2	Asia	Ph. companies	Canagliflozin 100 mg/canagliflozin 300 mg/placebo	E + S
Rosenstock et al. [29]	54.9	International	Ph. companies	Canagliflozin 100 mg + metformin/canagliflozin 300 mg + metformin/canagliflozin 100 mg/canagliflozin 300 mg/metformin	E + S
Kadowaki et al. [30]	57.2	Asia	Ph. companies	Canagliflozin 100 mg + teneligliptin 20 mg/placebo + teneligliptin 20 mg	E + S
Lingvay et al. [31]	56.6	International	Ph. companies	Canagliflozin 300 mg/semaglutide 10 mg	E + S

*Dapagliflozin*					
Bailey et al. [32]	53.9	International	Ph. companies	Dapagliflozin 2.5 mg/dapagliflozin 5 mg/dapagliflozin 10 mg/placebo	E + S
Ferrannini et al. [33]	52.0	International	Ph. companies	Dapagliflozin 10 mg/dapagliflozin 2.5 mg/dapagliflozin 5 mg/placebo	E + S
Nauck et al. [34]	58.5	International	Ph. companies	Dapagliflozin 2.5 → 10 mg/glipizide 5 → 20 mg	E + S
Strojek et al. [35]	59.8	International	Ph. companies	Dapagliflozin 2.5 mg/dapagliflozin 5 mg/dapagliflozin 10 mg/placebo	E + S
Bailey et al. [36]	52.5	International	Ph. companies	Dapagliflozin 1 mg/dapagliflozin 2.5 mg/dapagliflozin 5 mg/placebo	E + S
Bolinder et al. [37]	60.7	Europe	Ph. companies	Dapagliflozin 10 mg/placebo	E + S
Jabbour et al. [38]	54.9	International	Ph. companies	Dapagliflozin 10 mg/placebo	E + S
Ji et al. [39]	51.3	Asia	Ph. companies	Dapagliflozin 5 mg/dapagliflozin 10 mg/placebo	E + S
Kaku et al. [40]	58.8	Asia	Ph. companies	Dapagliflozin 5 mg/dapagliflozin 10 mg/placebo	E + S
Cefalu et al. [41]	62.9	International	Ph. companies	Dapagliflozin 10 mg/placebo	E + S
Mathieu et al. [42]	55.1	International	Ph. companies	Dapagliflozin 10 mg + saxagliptin 5 mg + metformin 500 mg/placebo + saxagliptin 5 mg + metformin 500 mg	E + S
Matthaei et al. [43]	61.0	International	Ph. companies	Dapagliflozin 10 mg/placebo	E + S
Schumm-Draeger et al. [44]	57.7	International	Ph. companies	Dapagliflozin 2.5 mg/dapagliflozin 5 mg/dapagliflozin 10 mg/placebo	E + S
Rosenstock et al. [45]	54.0	International	Ph. companies	Dapagliflozin 10 mg + saxagliptin 5 mg/dapagliflozin 10 mg + placebo/saxagliptin 5 mg + placebo	E + S
Frias et al. [46]	54.3	International	Ph. companies	Exenatide 2 mg + dapagliflozin 10 mg/exenatide 2 mg + placebo/dapagliflozin 10 mg + placebo	E + S
Weber et al. [47]	56.5	International	Ph. companies	Dapagliflozin 10 mg/placebo	E + S
Yang et al. [48]	53.8	Asia	Ph. companies	Dapagliflozin 5 mg/dapagliflozin 10 mg/placebo	E + S
Fioretto et al. [49]	65.8	International	Ph. companies	Dapagliflozin 10 mg/placebo	E + S
Yang et al. [50]	57.5	Asia	Ph. companies	Dapagliflozin 10 mg/placebo	E + S
Rosenstock et al. [51]	56.7	International	Ph. companies	Metformin + dapagliflozin 5 mg + saxagliptin 5 mg/metformin + dapagliflozin 5 mg/metformin + saxagliptin 5 mg	E + S
Vilsbøll et al. [52]	55.5	International	Ph. companies	Dapagliflozin 10 mg + saxagliptin 5 mg/insulin glargine	E + S
Sahay et al. [53]	49.0	Asia	Ph. companies	Dapagliflozin 10 mg + sitagliptin 100 mg + metformin 1000 mg/sitagliptin 100 mg + metformin 1000 mg/dapagliflozin 10 mg + metformin 1000 mg	E + S

*Empagliflozin*					
Häring et al. [54]	57.1	International	Ph. companies	Empagliflozin 10 mg/empagliflozin 25 mg/placebo	E + S
Roden et al. [55]	55.0	International	Ph. companies	Empagliflozin 10 mg/empagliflozin 25 mg/sitagliptin 100 mg/placebo	E + S
Barnett et al. [56]	62.6	International	Ph. companies	Empagliflozin 10 mg/empagliflozin 25 mg/Placebo	E + S
Kovacs et al. [57]	54.5	International	Ph. companies	Empagliflozin 10 mg/empagliflozin 25 mg/placebo	E + S
Ridderstråle et al. [58]	55.9	International	Ph. companies	Empagliflozin 25 mg/glimepiride 1–4 mg	E + S
Araki et al. [59]	60,1	Asia	Ph. companies	Empagliflozin 10 mg/empagliflozin 25 mg/placebo	E + S
DeFronzo et al. [60]	56.2	International	Ph. companies	Empagliflozin 25 mg + linagliptin 5 mg/empagliflozin 10 mg + linagliptin 5 mg/empagliflozin 25 mg/empagliflozin 10 mg/linagliptin 5 mg	E + S
Lewin et al. [61]	54.6	International	Ph. companies	Empagliflozin 25 mg + linagliptin 5 mg/empagliflozin 10 mg + linagliptin 5 mg/empagliflozin 25 mg/empagliflozin 10 mg/linagliptin 5 mg	E + S
Tikkanen et al. [62]	60.2	International	Ph. companies	Empagliflozin 10 mg/empagliflozin 25 mg/placebo	E + S
Hadjadj et al. [63]	52.3	International	Ph. companies	Empagliflozin 12.5 mg + metformin 1000 mg/empagliflozin 12.5 mg + metformin 500 mg/empagliflozin 5 mg + metformin 1000 mg/empagliflozin 5 mg + metformin 500 mg/empagliflozin 25 mg/empagliflozin 10 mg/metformin 1000 mg/metformin 500 mg	E + S
Søfteland et al. [64]	54.9	International	Ph. companies	Empagliflozin 10 mg + linagliptin 5 mg/empagliflozin 25 mg + linagliptin 5 mg/placebo + linagliptin 5 mg	E + S
Kawamori et al. [65]	59.9	Asia	Ph. companies	Empagliflozin 10 mg + linagliptin 5 mg/empagliflozin 25 mg + linagliptin 5 mg/placebo + linagliptin 5 mg	E + S
Ferdinand et al. [66]	56.8	United States	Ph. companies	Empagliflozin adjusting the dose from 10 mg to 25 mg/placebo	E + S
Rodbard et al. [67]	58.0	International	Ph. companies	Empagliflozin 25 mg/semaglutide 14 mg	E + S
Ji et al. [68]	60.2	Asia	Ph. companies	Empagliflozin 10 mg/empagliflozin 25 mg/placebo	E + S

*Enavogliflozin*					
Kim et al. [69]	58.6	Asia	Ph. companies	Enavogliflozin 0.3 mg/dapagliflozin 10 mg	E + S

*Ertugliflozin*					
Terra et al. [70]	56.4	International	Ph. companies	Ertugliflozin 5 mg/ertugliflozin 15 mg/placebo	E + S
Dagogo-Jack et al. [71]	59.1	International	Ph. companies	Ertugliflozin 5 mg/ertugliflozin 15 mg/placebo	E + S
Grunberger et al. [72]	67.3	International	Ph. companies	Ertugliflozin 5 mg/ertugliflozin 15 mg/placebo	E + S
Hollander et al. [73]	58.2	International	Ph. companies	Ertugliflozin 15 mg/ertugliflozin 5 mg/glimepiride 3 mg	E + S
Miller et al. [74]	55.6	International	Ph. companies	Ertugliflozin 5 mg + sitagliptin 100 mg/ertugliflozin 15 mg + sitagliptin 100 mg/placebo	E + S
Pratley et al. [75]	55,1	International	Ph. companies	Ertugliflozin 5 mg/ertugliflozin 15 mg/sitagliptin 100 mg/ertugliflozin 5 mg + sitagliptin 100 mg/ertugliflozin 15 mg + sitagliptin 100 mg	E + S
Rosenstock et al. [76]	56.6	International	Ph. companies	Ertugliflozin 5 mg/ertugliflozin 15 mg/placebo	E + S
Ji et al. [77]	56.5	Asia	Ph. companies	Placebo/ertugliflozin 5 mg/ertugliflozin 15 mg	E + S

*Ipragliflozin*					
Kashiwagi et al. [78]	59.7	Asia	Ph. companies	Ipragliflozin 50 mg/placebo	E + S
Kashiwagi et al. [79]	59.5	Asia	Ph. companies	Ipragliflozin 50 mg/placebo	E + S
Lu et al. [80]	53.7	Asia	Ph. companies	Ipragliflozin 50 mg/placebo	E + S
Han et al. [81]	57.5	Asia	Ph. companies	Ipragliflozin 50 mg/placebo	E + S
Shestakova et al. [82]	58.5	Europe	Ph. companies	Ipragliflozin 50 mg/placebo	E + S
Seino et al. [83]	55.5	Asia	Ph. companies	Sitagliptin 50 mg + ipragliflozin 50 mg/ipragliflozin 50 mg + placebo	E + S

*Luseogliflozin*					
Seino et al. [84]	59.3	Asia	Ph. companies	Luseogliflozin 2.5 mg/placebo	E + S
Shestakova et al. [85]	58.5	Europe	Ph. companies	Luseogliflozin 2.5 mg/luseogliflozin 5 mg/luseogliflozin 10 mg/placebo	E + S

*Remogliflozin*					
Dharmalingam et al. [86]	50.7	Asia	Ph. companies	Remogliflozin 100 mg/remogliflozin 250 mg/canagliflozin 10 mg	E + S

*Tofogliflozin*					
Kaku et al. [87]	57.2	Asia	Ph. companies	Tofogliflozin 10 mg/tofogliflozin 20 mg/tofogliflozin 40 mg/placebo	E + S

^a^Ph. companies = pharmaceutical companies.

^b^E + S = efficacy + safety.

**Table 3:** Proportion of women and other features of gender assessment.

**Study (first author)**	**First author gender**	**Corresponding author gender**	**Last author gender** ^**a**^	**Total**	**Women**	**% women**	**F-Particip**	**Sex bias**
*Bexagliflozin*								
Allegretti et al. [18]	M	M	F	312	116	37.2	0.372	−0.108

*Canagliflozin*								
Bode et al. [19]	M	M	M	714	318	44.54	0.445	−0.035
Cefalu et al. [20]	M	M	M	1450	694	47.86	0.479	−0.001
Lavalle-González et al. [21]	M	M	M	1284	679	52.88	0.529	0.049
Schemtaner et al. [22]	M	M	M	755	333	44.11	0.441	−0.039
Stenlöf et al. [23]	M	M	M	584	326	55.82	0.558	0.078
Wilding et al. [24]	M	M	M	469	230	49.04	0.490	0.010
Yale et al. [25]	M	M	M	269	106	39.41	0.394	−0.086
Forst et al. [26]	M	M	M	342	126	36.84	0.368	−0.112
Inagaki et al. [27]	M	M	M	271	80	29.52	0.295	−0.185
Ji et al. [28]	M	M	M	676	314	46.45	0.464	−0.016
Rosenstock et al. [29]	M	M	F	1186	617	52.02	0.520	0.040
Kadowaki et al. [30]	M	F	F	138	31	22.46	0.225	−0.255
Lingvay et al. [31]	F	F	M	788	364	46.19	0.462	−0.018

*Dapagliflozin*								
Bailey et al. [32]	M	M	M	546	254	46.52	0.465	−0.015
Ferrannini et al. [33]	M	M	M	558	282	50.54	0.505	0.025
Nauck et al. [34]	M	M	M	801	360	44.94	0.449	−0.031
Strojek et al. [35]	M	M	M	592	307	51.86	0.519	0.039
Bailey et al. [36]	M	M	M	282	141	50.00	0.500	0.020
Bolinder et al. [37]	M	M	M	180	80	44.44	0.444	−0.036
Jabbour et al. [38]	M	M	M	447	202	45.19	0.452	−0.028
Ji et al. [39]	M	M	M	393	136	34.61	0.346	−0.134
Kaku et al. [40]	M	M	F	261	106	40.61	0.406	−0.074
Cefalu et al. [41]	M	M	M	914	290	31.73	0.317	−0.163
Mathieu et al. [42]	F	F	M	320	174	54.4	0.544	0.064
Matthaei et al. [43]	M	M	M	216	110	50.93	0.509	0.029
Schumm-Draeger et al. [44]	F	F	M	399	220	55.14	0.551	0.071
Rosenstock et al. [45]	M	M	M	534	266	49.81	0.498	0.018
Frias et al. [46]	M	M	M	685	357	52.12	0.521	0.041
Weber et al. [47]	M	M	F	449	202	44.99	0.450	−0.030
Yang et al. [48]	M	M	F	444	203	45.72	0.457	−0.023
Fioretto et al. [49]	F	F	M	321	139	43.30	0.433	−0.047
Yang et al. [50]	F	F	F	272	142	52.21	0.522	0.042
Rosenstock et al. [51]	M	M	M	870	419	48.2	0.482	0.002
Vilsbøll et al. [52]	F	F	M	643	296	46.03	0.460	−0.020
Sahay et al. [53]	M	M	F	415	166	40.00	0.400	−0.080

*Empagliflozin*								
Häring et al. [54]	M	M	M	666	327	49.10	0.491	0.011
Roden et al. [55]	M	M	M	899	348	38.71	0.387	−0.093
Barnett et al. [56]	M	M	M	290	113	38.97	0.390	−0.090
Kovacs et al. [57]	M	M	M	498	257	51.61	0.516	0.036
Ridderstråle et al. [58]	M	M	M	1545	692	44.79	0.448	−0.032
Araki et al. [59]	F	F	M	1160	325	28.02	0.280	−0.200
DeFronzo et al. [60]	M	M	M	674	312	46.29	0.463	−0.017
Lewin et al. [61]	M	M	M	667	308	46.18	0.462	−0.018
Tikkanen et al. [62]	M	M	M	823	328	39.85	0.399	−0.081
Hadjadj et al. [63]	M	M	M	1380	592	42.90	0.429	−0.051
Søfteland et al. [64]	M	M	M	327	130	39.76	0.398	−0.082
Kawamori et al. [65]	M	F	M	275	61	22.18	0.222	−0.258
Ferdinand et al. [66]	M	M	M	150	71	47.33	0.473	−0.007
Rodbard et al. [67]	F	F	M	821	406	49.5	0.495	0.015
Ji et al. [68]	M	M	M	219	100	45.66	0.457	−0.023

*Enavogliflozin*								
Kim et al. [69]	M	M	M	270	129	47.78	0.478	−0.002

*Ertugliflozin*								
Terra et al. [70]	F	F	M	461	200	43.38	0.434	−0.046
Dagogo-Jack et al. [71]	M	M	M	462	199	43.07	0.431	−0.049
Grunberger et al. [72]	M	M	M	467	236	50.54	0.505	0.025
Hollander et al. [73]	F	M	M	1325	683	51.54	0.515	0.035
Miller et al. [74]	M	M	M	291	124	42.61	0.426	−0.054
Pratley et al. [75]	M	M	M	1232	568	46.10	0.461	−0.019
Rosenstock et al. [76]	F	F	F	621	333	53.62	0.536	0.056
Ji et al. [77]	M	M	M	506	225	44.46	0.445	−0.035

*Ipragliflozin*								
Kashiwagi et al. [78]	M	F	M	240	82	34.17	0.342	−0.138
Kashiwagi et al. [79]	M	M	M	129	39	30.23	0.302	−0.178
Lu et al. [80]	M	M	M	170	93	54.71	0.547	0.067
Han et al. [81]	F	M	M	139	70	50.36	0.504	0.024
Shestakova et al. [82]	F	F	M	165	95	57.58	0.576	0.096
Seino et al. [83]	M	M	M	141	42	29.79	0.298	−0.182

*Luseogliflozin*								
Seino et al. [84]	M	M	M	158	42	26.58	0.266	−0.214
Shestakova et al. [85]	F	F	M	328	212	64.63	0.646	0.166

*Remogliflozin*								
Dharmalingam et al. [86]	F	M	M	611	271	44.35	0.444	−0.036

*Tofogliflozin*								
Kaku et al. [87]	M	M	M	229	76	33.19	0.332	−0.148

^a^F = female; M = male.

**Table 4:** Proportion of women and other features of gender assessment according to the different subgroups.

**Subgroup**	**Studies**	**Representation of women**				
	**N**	**N** ** patients**	**N** ** women**	**Percentage**	**F-Particip**	**Sex bias**
Total	70	38,119	17,275	45.31	0.453	−0.027
Drug						
Bexagliflozin	1	312	116	37.2	0.372	−0.108
Canagliflozin	13	8926	4218	47.3	0.473	−0.007
Dapagliflozin	22	10,542	4852	46.0	0.460	−0.020
Empagliflozin	15	10,394	4370	42.0	0.420	−0.060
Enavogliflozin	1	270	129	47.8	0.478	−0.002
Ertugliflozin	8	5365	2568	47.90	0.479	−0.001
Ipragliflozin	6	984	421	42.80	0.428	−0.052
Luseogliflozin	2	486	254	52.3	0.523	0.043
Remogliflozin	1	611	271	44.4	0.444	−0.036
Tofogliflozin	1	229	76	33.2	0.332	−0.148
Geography						
Asia	21	7117	2733	38.40	0.384	−0.096
Europe	3	673	387	57.50	0.575	0.095
International	45	30,179	14,084	46.70	0.467	−0.013
United States	1	150	71	47.3	0.473	−0.007
Date of publication						
2010–2015	37	21,235	9383	44.20	0.442	−0.038
2016–2023	33	16,884	7892	46.70	0.467	−0.013
Female leadership in authorship (first, corresponding, or last author), female/male						
3/0	2	893	475	53.20	0.532	0.052
2/1	11	5544	2462	44.40	0.444	−0.036
1/2	11	5657	2577	45.60	0.456	−0.024
0/3	46	26,025	11,761	45.20	0.452	−0.028
Sample size						
*N* 0–200	9	1370	563	41.1	0.410	−0.07
*N* 201–400	21	6127	2623	42.8	0.428	−0.052
*N* 401–700	23	12,675	6073	47.90	0.479	−0.001
*N* 701–1000	9	7385	3166	42.90	0.429	−0.051
*N* + 1000	8	10,562	4850	45.90	0.459	−0.021

## Data Availability

The data supporting this study's findings are available from the corresponding author upon reasonable request.
